# Interaction of HERVs with PAMPs in Dysregulation of Immune Response Cascade Upon SARS-CoV-2 Infections

**DOI:** 10.3390/ijms252413360

**Published:** 2024-12-12

**Authors:** Marijana Turčić, Sandra Kraljević Pavelić, Dragan Trivanović, Krešimir Pavelić

**Affiliations:** 1 Teaching Institute of Public Health of Primorsko-Goranska County, Krešimirova 52a, 51000 Rijeka, Croatia; turcic.m.ri@gmail.com; 2 Faculty of Health Studies, University of Rijeka, Ulica Viktora Cara Emina 5, 51000 Rijeka, Croatia; 3 Faculty of Medicine, Juraj Dobrila University of Pula, Zagrebačka 30, 52100 Pula, Croatia; dtrivanovic@obpula.hr; 4 Opća Bolnica Pula, Santoriova Ul. 24a, 52100 Pula, Croatia; 5 International Academy of Science, Arts and Religion, Radnička Cesta, 71000 Sarajevo, Bosnia and Herzegovina

**Keywords:** SARS-CoV-2, spike protein, HERV, immunity, COVID-19

## Abstract

Human endogenous retroviruses (HERVs) are genomic fragments integrated into human DNA from germline infections by exogenous retroviruses that threatened primates early in their evolution and are inherited vertically in the germline. So far, HERVs have been studied in the context of extensive immunopathogenic, neuropathogenic and even oncogenic effects within their host. In particular, in our paper, we elaborate on the aspects related to the possible correlation of transposable HERV elements’ activation and SARS-CoV-2 spike protein’s presence in cells of COVID-19 patients or upon COVID-19 vaccination with implications for natural and adaptive immunity. In particular, the release of cytokines TNF-α, IL-1β and IL-6 occurs in such cases and plays a notable role in sustaining chronic inflammation. Moreover, well-known interindividual variations of HERVs might partially account for the interpersonal variability of COVID-19 symptoms or unwanted events post-vaccination. Accordingly, further studies are required to clarify the SARS-CoV-2 spike protein’s role in triggering HERVs.

## 1. Introduction

**Figure 1:**
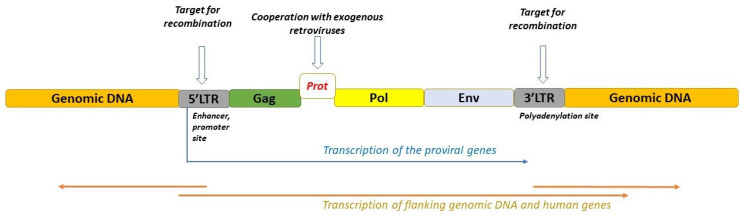
Schematic representation of the HERV element in human gene expression. The majority of HERV elements are solitary LTRs, arising due to homologous recombination between the identical long terminal repeats flanking proviral genes *Gag*, *Prot*, *Pol* and *Env*. Within the LTR sequences, polyadenylation signals, enhancer and promoter elements are present. These can initiate transcription of the flanking genomic loci. Courtesy adapted from [9].

Human endogenous retroviruses (HERVs) are genomic fragments integrated into human DNA from germline infections by exogenous retroviruses that threatened primates early in their evolution and are inherited vertically in the germline [1]. HERVs occupy about 8% of the human genome and have a high number of accumulated mutations. This is why the majority of these elements do not have open reading frames (ORFs) and cannot replicate. Accordingly, HERVs are not infectious but during the recombination between homologous long terminal repeats (LTRs), solitary LTR may occur. The exception is HERV-K elements that have higher activity and may transmit viral RNA to other cells [2,3,4,5]. HERVs are now usually classified according to the sequence comparison with animal retroviruses. Class I HERVs have homology with the genus Gammaretrovirus (families HERV-F, H, I, E, R, P, T, W, ERV-FTD, FRD), class II HERVs have homology with the genus Betaretrovirus (family HERV-K) and class III HERVs have homology with the genus Spumavirus-related (family HERV-L) [4,5,6]. A complete HERV element is about 9.5 kb in length, flanked by two LTRs, with regions including cis-acting elements and promoters that control gene expression, and is composed of four essential viral genes: *gag*, *prot*, *pol* and *env* [7,8]. The *gag* gene codes a polyprotein that, digested by viral proteases, turns into main structural capsid and nucleocapsid proteins which are engaged in the packaging of viral particles. The *pro* gene codes the protease for the processing of viral polypeptides, while *pol* gene codes reverse transcriptase and integrase proteins engaged in DNA synthesis and inclusion into the host genome. The *env* gene codes envelope proteins, such as surface and transmembrane envelope proteins, which are accountable for fusion and receptor recognition [8,9,10] (Figure 1).

However, during the evolution process, most integrated HERVs have been disrupted, and are mainly found in the heterochromatin, thus being suppressed by epigenetic silencing due to their harmful potential to the host cell [8]. Likewise, since HERVs have been vertically transmitted through generations, this resulted in numerous interindividual variations, e.g., polymorphism, unfixed copies and copy number variations [4,5,11]. In addition, several HERVs were naturalized to assist in some physiological functions during early human development. Later in the fetus and afterward in adulthood, these proteins are epigenetically silenced [12]. For example, syncytin-1 and syncytin-2, HERV-W envelope glycoproteins (ENV) and HERV-FRD are expressed in the placenta and play a central role in human embryogenesis as they are actively involved in the formation of the syncytiotrophoblast layer of the placenta [7]. HERV *gag* proteins are engaged in human brain development and architecture, and they also have a crucial role in the dispersion of endogenous retroviruses (ERV) in the evolution and formation of natural immune networks in mammalians [13,14]. Yet, it has been well documented that there are several external and internal factors like some viral and bacterial infections, cytokines, steroid hormones, heavy metals, UVB irradiation and exposure to extremely low-frequency electromagnetic fields (ELF-EMFs) that are able to reactivate these dormant HERV elements with diverse outcomes, depending on the triggering factor and receptive cells or tissues [10,15,16,17]. Hence, HERV activity and accordingly the host immune response differ and depend on interindividual variations and age, as well as comorbidities of the host [5,11,18].

## 2. The Role of HERVs in Disease

HERVs can impact the host through their regulatory signals that affect the expression of cellular genes, particularly their abundant production of RNA transcripts. Since these RNA transcripts contain viral signatures, they can be recognized as pathogen-associated molecular patterns (PAMPs) by native immune pathways [19]. These can trigger a cascade reaction leading to nuclear induction of the interferon-I (IFN-I) pathway and consequently the production of inflammatory cytokines. The latter are correlated with the onset and clinical symptoms of several inflammatory and autoimmune diseases (e.g., multiple sclerosis (MS), rheumatoid arthritis), dermatoses (e.g., lichen planus), endocrine disorders (e.g., type 1 diabetes), psychiatric diseases (e.g., bipolar disorders, schizophrenia), obstetrical disorders (e.g., preeclampsia) and infectious diseases (e.g., acquired immunodeficiency syndrome), but also in a number of malignancies (e.g., leukemia, hepatocellular, pancreatic, breast, ovarian and prostate cancer, germ cell tumors and Kaposi’s sarcoma) [10,11,15,16,19,20]. Epstein-Barr Virus (EBV), known to be among the risk factors for the subsequent development of MS, triggers upregulation in HERV-K18 and another six HERV loci. Consequently, a generally higher frequency of circulating HERV-W ENV was also found in patients with MS [21]. Namely, HERV-W encodes an immunopathogenic and neurotoxic HERV-W ENV which induces proinflammatory responses through the toll-like receptor 4 pathway (TLR-4) [15]. Such abnormal HERV expression can become self-sustained and thus generate chronic protein expression, e.g., with cytokine-mediated feedback loops, in affected tissue. This has also been reported to be the case in brain lesions with lifelong expansion in patients with MS [22,23]. Hence, a humanized monoclonal antibody targeting the HERV-W ENV has been developed and is recommended as an innovative immunotherapeutic approach for treating MS [24]. In schizophrenia and bipolar disorders, increased retroviral HERV-W family RNA was also detected. Also, HERVs, triggered by Kaposi’s sarcoma-associated herpesvirus (KSHV), are able to activate multiple oncogenic signaling pathways that promote the proliferation of cancer cells and suppress their differentiation and apoptosis. Importantly, activation of the extracellular signal-regulated kinase (ERK) pathway in cancer underlies the HERV ENVs’ immunosuppressive properties and promotion of carcinogenesis [25,26,27]. Still, in some cases, HERVs may also exert the opposite role in cancer patients and activate immunity and inhibit cancer cell growth. For example, in breast, ovarian and colorectal cancer, cytotoxic T cell-mediated immunity has been reported to be induced by HERV ENVs [10]. Indeed, tumors often produce both internal and tumor cell surface antigens. Tertiary Lymphoid Structures (TLSs), for example, correlate with improved patient survival and response to immune checkpoint inhibitors (ICI), but a clear mechanism of this effect is not explained [28,29]. Interestingly, these tumor-associated antigens (TAAs) also include antigens derived from endogenous retroviruses (ERVs). These show low expression in healthy tissues but are often upregulated in cancer cells due to insufficient recognition and immunological tolerance, respectively [30,31]. The discovery of HERV antigens’ immunogenicity provides an exciting base for the explanation of some aspects of the carcinogenesis process. Still, the consequence or protection of B cell antigen production has not been fully elucidated so far [32,33,34].

## 3. Lung Cancer and HERVs: lncRNA Role in Disease Pathogenesis

Lung cancer is one of the most prevalent and deadly cancers worldwide but is also characterized as an immunologically hot cancer with a high mutational burden, as described in melanomas. Hence, it is not surprising that the role of ERV antigens has been studied in lung cancer [35,36]. Smoking was shown to induce HERV-K (HML-2 and 6) pol expression in multiple cancer and precancer tissues but there is also evidence that HERV-K (HML-2) solo-LTR (1p13.2) homozygosity in “women never-smokers” is statistically associated with increased susceptibility to lung adenocarcinoma [37,38]. HERV-K (HML-2) envelope-reactive antibodies have also been identified upon SARS-CoV-2 infection in healthy individuals as well as patients with systemic lupus erythematosus (SLE) [39,40]. Provided research data show that establishing an unequivocal link between smoking and HERV antigen expression is not possible based on the factor of “smoking” alone, and the data point to the activation of HERV elements and the formation of antigens as a smoking-independent process. In recent years, endogenous retroviral-associated adenocarcinoma EVADR long non-coding ribonucleic acid (lncRNA) has been researched as a relevant lung carcinogenesis mechanism. Moreover, EVADR lncRNA was detected in over 20% of patients with lung adenocarcinoma but undetected in normal lung tissues [41]. EVADR expression in multiple cancers indicated a significant connection with decreased survival rates [41,42]. Altogether, tumor antigen expression opens up the question of the exact role of HERV antibodies and the immunotherapy effect in cancer patients. Ng et al., for example, investigated lung B cell responses in patients from the project TRACERx 421 and other lung cancer databases. Their result proves that human and mouse lung adenocarcinomas pull out the production of tumor-binding antibodies and, moreover, identified endogenous retrovirus (ERV) envelope glycoproteins as a strong anti-tumor antibody target, which probably requires CXCL13-dependent TLS formation [43]. They elegantly proved that B cell responses contribute to therapy response through the production of protective antibodies and by establishing ERV envelope glycoproteins as relevant tumor antigens. Further research in tumor antigens, HERV antigens and immunotherapy effects will be critical to inform the use of B cell mechanisms in predicting and enhancing tumors’ susceptibility to immunotherapy.

In summary, HERVs are known to be able to activate extensive immunopathogenic, neuropathogenic and even oncogenic effects within their host. Expression of HERV ENVs in adult cells is not the only one that is a usual consequence of environmental triggers but is certainly among those events that are harmful to the host, as reported in a number of human disorders and diseases.

## 4. Role of HERVs in Natural Immunity Response

**Figure 2:**
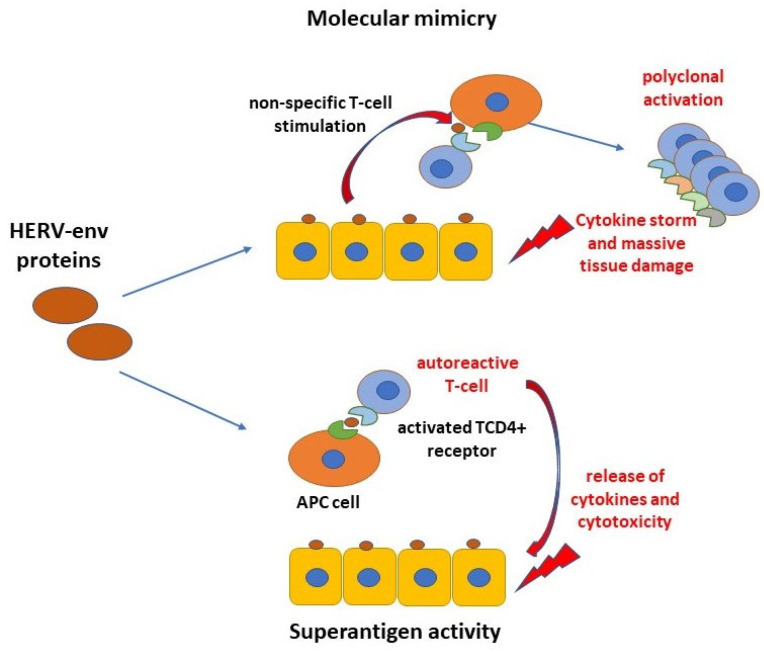
Schematic representation of the HERV-env protein’s possible role in autoimmunity. The HERV-derived Env proteins stimulate host immunity through molecular mimicry and superantigen activity. In molecular mimicry, the antigen-presenting cells (APCs) present endogenous Env antigens from healthy cells to previously activated T cells by viral env proteins (cross-reactivity). This triggers autoimmunity mechanisms through the release of proinflammatory cytokines and adaptive responses which ends with tissue injury of env-producing cells. In the mechanism of superantigen, non-specific stimulation of T lymphocytes occurs by HERV-env. Polyclonal expansion of reactive T cells is initiated and massive cytokine release occurs. This process results in extensive tissue damage and often systemic life-threatening manifestations due to organ failure. Adapted from [50].

Natural immunity is the basic defense line of the organism against infections due to its ability to recognize the existence of PAMPs through pattern recognition receptors (PRRs) that alert the organism of the infection and cellular damage or stress. Over the past few decades, thanks to advanced sequencing technology, HERVs have been documented and studied in many diseases and identified as main facilitators and/or activators of natural immunity-related genes. These genes are considered to play a significant role in natural immunity response, especially by initiating IFN signaling. HERVs exert their activity via the functional impact of their four crucial viral components (HERV-derived RNAs; HERV-derived RNA, namely deoxyribonucleic acid (DNA), duplexes and cDNA; HERV-derived proteins and ribonucleoprotein complexes; and HERV LTRs). [27]. Accordingly, several studies found that HERVs are located close to natural immunity-related, IFN-inducible genes [44,45,46]. Activated by environmental provocation or cellular stress, HERV-derived DNA, RNA and proteins can be recognized by three different types of PRRs: TLRs, retinoic acid-inducible (RIG)-I-like receptors (RLRs) and cyclic GMP-AMP synthase (cGAS). Following PRR stimulation, various signaling pathways are triggered, resulting in the activation of natural immune response via the transcription of nuclear factors NF-kB and IFN regulatory factors, IRFs 3/7. Their activation leads to the production of inflammatory cytokines, which stay for an anti-viral state that imitates the reaction of the host to an exogenous infection. Specifically, the HERV-W family, interacting with TLRs, generates a significant proinflammatory response and the release of cytokines TNF-α, IL-1β and IL-6, respectively [47]. Notably, these proinflammatory cytokines additionally boost the HERV activity. Through such proinflammatory cytokine actions, as well as interferon signaling feedback loops, HERV expression remains upregulated, thereby continually hyperactivating the natural immunity response [20,46,48]. Accordingly, the HERV mechanism and involvement in the pathogenesis of diseases is probably based on underlying chronic inflammation and autoimmunity. For example, in the context of COVID-19-diagnosed patients, a retroelement MIRb has been found to be activated by SARS-CoV-2 in vitro. MIRb seems to exert its activity as a promoter for the synthesis of a new isoform of ACE2, termed MIRb-ACE2, that is inducible by IFN [49]. According to the authors of this study, this transcript is formed by splicing between exons of *ACE2* and an *LTR16A1* retroelement, integrated in intron 9 of the *ACE2* locus. It has been also established that the primary receptor of SARS-CoV-2 is ACE2 that is truncated and consequently unstable in the form of MIRb-ACE2. Such a form may hamper the SARS-CoV-2 entering the cell and compromise its binding to the receptor. This is only one example of the solitary retroviral LTRs involved in the regulation of an antiviral response. Other HERV elements, as stated previously, may play important roles as well. For example, HERV products known as HERV-env may exert a pro-oncogenic role besides their physiological roles. In particular, the fusogenic and immunosuppressive activities of the HERV-K(HML2) sequences can underly the malignant transformation of cells through their accessory proteins Np9 and Rec, better known as HERV-derived oncogenes [50]. Indeed, the HERV-env are known to generally have the ability to induce inflammation and cytotoxicity [51] but also activate the immune response as superantigens that directly stimulate T lymphocytes and strong cytokine release that can cause massive tissue damage [50] (Figure 2).

## 5. Activation of HERV in COVID-19 Patients

Severe acute respiratory syndrome coronavirus (SARS-CoV-2) is described in the scientific literature as a betacoronavirus from the Coronavirinae subfamily of the Coronaviridae and is still subject to discussions on its origin and thus sometimes termed as a viroid [52,53,54,55]. The main aspect of SARS-CoV-2’s presence in vivo in the clinical state, termed human coronavirus disease 2019 (COVID-19), is characterized by an intense hyperinflammatory reaction, also called “cytokine storm”, that in some cases leads to multiple organ dysfunction and death, or durable and severe continuance to post-acute problems termed “long-COVID syndrome”. For instance, the latest studies indicate that about 32% to 87% of COVID-19 patients develop post-acute long COVID syndrome [56,57,58]. The precise mechanism of how SARS-CoV-2 or its components may activate hyperinflammation is still weakly known. It has been observed that in some cases the viral particles or the spike components are not quickly eliminated from the organism, resulting in post-acute persistence in the blood. This situation may not be ruled out for COVID-19 post-vaccination states as well. This is a relevant possibility, as due to its common antigenic epitopes with human molecular chaperons, it appears that the SARS-CoV-2 S protein can directly damage tissues and organs. This damage occurs by triggering adaptive immunity to autoimmunity by developing a large number of different autoantibody classes versus endothelial cells, as it was observed in multisystem inflammatory syndrome (MIS) in children, as well as in patients developing de novo or exacerbating preceding autoimmune diseases [59,60,61]. Yet intriguingly, the latest study revealed that post-acute long COVID is not correlated with autoantibodies, but with upraised proinflammatory cytokines interleukin 1β (IL-1β), interleukin 6 (IL-6) and TNF-α, indicating them as intermediates of an evolving SARS-CoV-2-controlled immunity response to a persisting virus or its antigens, and/or constant reprogramming of immune cells [62].

Dysregulation of natural and adaptive immunity response also plays a crucial role in the clinical outcomes of COVID-19 patients which may be partially correlated with observed increased expression of HERVs among these patients. Indeed, a number of studies showed that SARS-CoV-2 presence modulates the transcription of HERV loci, thereby affecting the process of viral penetration, the outbreak of symptoms, clinical stages and the disease severity [15,63]. HERVs’ activation and their recognition as PAMPs by cellular PRRs induce natural immunity response, support the correlation of HERV activation and hyperinflammatory reaction which is observed in some COVID-19 patients. This fact may be highly relevant for the understanding and management of this pathology. In particular, published results show an association between SARS-CoV-2 infection and increased expression of HERV-W, HERV-K, TNF-α, proinflammatory cytokines IL-1β, IL-6, interleukin 17 (IL-17), monocyte chemoattractant protein-1 (MCP-1), TRL-3, TRL-7 and interferon ϒ (INF-ϒ) [5,47]. Yet, disease severity and lethal outcome are correlated with the increased expression of IL-6 and TNF-α. Thus, IL-6-focused treatment combined with corticosteroids, as well as TNF blockade, showed positive results in severe cases of acute COVID-19 [62,64,65]. Furthermore, it was found that levels of C-reactive protein (CRP), a component known to induce the output of proinflammatory cytokines, apoptosis and inflammation, have a positive correlation with the severity of COVID-19 [66,67]. This positive correlation was also reported in COVID-19 patients presenting with depression or psychotic symptoms, even without previous positive anamnesis of such disorders. Notably, the same correlation was found in patients suffering from schizophrenia, confirming the theory of HERV-W playing a significant role in sustaining chronic inflammation [68,69]. Recent studies also indicate that HERV-W ENV, being an underlying pathogenic factor correlated with disease severity, can explain the interpersonal variability in COVID-19 symptoms for HERVs’ well-known interindividual variations [24]. HERV-W ENV was found to be triggered, and therefore highly expressed in leukocytes, a significant number of CD4 T-lymphocytes, as well as B-lymphocytes of COVID-19 patients [64,70,71]. Since this HERV-W expression in T-cells was not observed in any other pathological state before, it is considered that the SARS-CoV-2 spike (S) protein is the one provoking its expression in those cells [15,16]. Moreover, whereas HERV-W ENV expression also corresponds with the T-cell differentiation markers and exhaustion, it may lead to lymphocyte deficiency, consequently resulting in lymphopenia, hyperneutrophilia and the hyperactivation of natural immunity [72,73,74]. HERV-W ENVs may be incorporated not only into the cell membranes but soluble HERV-W ENVs can also be released into the plasma. In fact, the percentage of HERV-W ENV-positive lymphocytes correlates with the presence of HERV-W ENV in plasma and with inflammatory markers and the severity of pneumonia that reflects the respiratory outcome in hospitalized patients [15,71]. Nevertheless, the mentioned HERV-W ENV expression observed in the blood of COVID-19 patients was also detected in tissue-infiltrated lymphoid cells within affected organs, including the cells of the central nervous system. Accordingly, HERV-W ENV expression is considered to play a significant role in neurological symptoms and cognitive impairment occurring and/or persisting during post-acute COVID, especially regarding previously well-known HERV-W ENV association with the microglia-driven pathogenesis of MS and the detection of a soluble HERV-W ENV in MS brain lesions [21,22,23,72,75,76]. Intriguingly, the existence of significantly increased HERV-W ENV in plasma was only detected in severe, hospitalized COVID-19 cases, and was not detected at all in healthy controls. However, it is still undetermined which HERV-W loci are triggered in COVID-19 since there are a minimum of 13 HERV-W loci encoding full-length ENV in the human genome [77]. Nevertheless, HERV-W ENV could still represent a biomarker of COVID-19 severity, as well as previously mentioned HERV-W expression in blood T lymphocytes.

On the contrary, HERV-R-ENV levels were found to be reduced both in cell-based models and COVID-19 patient samples upon SARS-CoV-2 infection in a recent study [78]. The authors point to the antiviral HERV-R-ENV role mediated by the ERK pathway. Accordingly, the SARS-CoV-2 antagonizes the antiviral function of HERV-R-ENV by upregulating the p38 MAPK activity and concomitantly downregulating the ERK activity. HERV-R-ENV has already been described as an immune system modulator as it may trigger inflammation but may also be involved in autoimmunity and malignant diseases [79].

## 6. Implications for HERV Elements Activation Upon COVID-19 Vaccination

Our immune system is prepared to react properly against SARS-CoV-2 particles as in other viral-induced states. In particular, innate immunity is crucial as a first-line defense against coronaviruses including SARS-CoV-2. The critical role in immune protection against severe COVID-19 disease is thus covered by the innate immune cells of the mucosal epithelia, including macrophages, monocytes, dendritic cells, neutrophils and innate lymphoid cells (ILCs) such as natural killer (NK) cells. The innate cells infected by the coronavirus activate the innate immune cells and the production of inflammatory cytokines and chemokines. Consequently, cell death of infected cells occurs. If this process is not controlled, systemic “cytokine storm” may occur, as already observed during COVID-19. Further on, lymphocytes react versus all regions of the SARS-CoV-2 proteome. Namely, CD8^+^ T lymphocytes eliminate infected cells and constrain infection, whereas CD4^+^ T lymphocytes provide signals needed for the antibody response. Therefore, the natural immunity response plays a significant role in containing SARS-CoV-2’s spread, manifested in asymptomatic and mild infections in children and young adults, respectively [80,81,82]. Yet, the onset of post-acute long COVID syndrome in certain patients indicates immune disturbances being a contributing factor in COVID-19 immunopathology of prolonged and inadequate immune response.

As mentioned, the SARS-CoV-2 S protein, a monomer on the surface of the SARS-CoV-2 and one of the four major SARS-CoV-2 proteins, is responsible for enabling host cell receptor recognition, viral attachment and viral cell entry into the host cell. This S protein is composed of two different attached subunits, S1 and S2. After binding to the ACE2 receptor on the surface of human cells, the S1 subunit is detached and released into the blood, while the S2 subunit interposes viral fusion and the entrance of the virus into the cell [54,55,83,84]. Interestingly, studies show that only the S protein is a potent PAMP, therefore able to be recognized by PRRs, resulting in arousing natural immunity response in COVID-19. Notably, SARS-CoV-2 S is able to cause additional harm by activating TLRs, particularly TLR2 at the surface of macrophages and monocytes. This results in activation of the NF-kB pathway with proinflammatory cytokine secretion even without entry of the whole viral particle in the cell [85,86]. In line with these mechanisms, it has been found that deceased COVID-19 patients manifested a massive influx of neutrophils and macrophages yet decreased levels of T cells in their blood, therefore indicating the connection between hyperactivation of natural immunity and COVID-19 pathogenesis [87]. Recent studies also described a positive correlation between high levels of S1 and poor outcomes in COVID-19. It is described that S1 alone could have a substantial role in activating inflammatory signaling, as well as sustaining the “cytokine storm” (TNF-α, IL-1β, IL-6), through activation of NF-kB and/or extracellular signal-regulated kinase 1/2 (ERK1/2) cascade in human peripheral blood mononuclear cells [88,89]. Moreover, S1 is able to attach to the surface glycoprotein neuropilin-1 (NRP-1), leading not only to infectivity augmentation but also to the deregulation of immune responses, angiogenesis and neuronal development [90]. Notably, due to its ability to generate a loss of human blood–brain barrier integrity, S1 activates microglia and thus the subsequent increased release of previously mentioned proinflammatory cytokines in the endothelium [91]. The latest suggests a possible mechanism regarding neurological symptoms in post-acute long COVID, as the latest data revealed the presence of S1 in the blood even up to 12 months, and in monocytes up to 15 months after the initial diagnosis in most patients with post-acute long COVID [92,93]. Importantly, many studies do not discriminate the state of S protein persistence in the body in those patients who received a COVID-19 vaccine in comparison with those who are not vaccinated. The current plausible conclusion points accordingly to an S protein long-persistence property in vivo which should be carefully evaluated for the safety aspects of any product based on the introduction of an S protein in the body or a genetic construct for the production of the S protein. In line with this concern, it has also been revealed that the SARS-CoV-2 S protein contains a hexapeptide pattern highly similar to inhibitory tyrosine-based immunoreceptor motif (ITIM) in a human Leukemia Inhibitory Factor Receptor (LIFR), a pneumonia protective IL-6 family cytokine receptor. Since functional ITIM sequences are located inside the cytoplasmic tag end of immunoreceptors, taking into consideration the capability of exogenously entered ITIM containing peptides to inhibit TRL-mediated ITIM-dependent signaling, could lead to the probability of virus derivative ITIMs to equivalently modulate inhibitory ITIM signaling, leading to host breakdown in self-tolerance [64,94]. This supports the idea that prolonged and inadequate immune response is related to S protein persistence and effects in those patients.

Interestingly, several previously conducted studies demonstrated that in vitro exposure of leukocytes to the SARS-CoV-2 S protein triggers a powerful and sustainable expression of HERV-W ENV in those cells. This activation appears without signs of leukocyte activation by SARS-CoV-2 and it happens prior to the expression of IL-6, the induction of which arises 24 h later [15,70]. In addition, the amount of HERV-W ENV messenger ribonucleic acid (mRNA) was found to be highly correlated with the amount of IL-6, an already noticed indicator of disease deterioration [95]. Interestingly, the recombinant trimeric SARS-CoV-2 S protein, along with undetectable HERV ribonucleic acid (RNA) or protein expression, has the ability to induce IL-6 production in native human peripheral blood mononuclear cells (PBMC), which confirms that SARS-CoV-2 S protein induces HERV activation, thus leading to hyperinflammatory and native immune reactions [70]. Due to HERVs’ ability to stimulate natural immunity response, as being recognized as PAMPs through PRRs, activation of HERVs by SARS-CoV-2 S seems to be yet another way for SARS-CoV-2 S protein to increase proinflammatory response and the release of certain cytokines (TNF-α, IL-1β, IL-6), already indicated as key intermediates in the immune pathogenesis in post-acute long COVID. Consequently, these inflammatory cytokines lead to increased HERV activity, generating chronic protein expression, e.g., with cytokine-mediated feedback loops, in affected tissue. For instance, TNF-α was shown to have the capability of increasing the RNA expression of HERV-W, HERV-K and HERV-H due to the translocation of NF-kB to the nucleus with consequent linking to parts presenting HERV LTRs. Furthermore, the increased levels of TLRs mRNA in COVID-19 and MIS in children correlate with HERV activation, probably due to HERVs’ capability to activate PRRs, and thus proinflammatory cytokine production [96]. The latest results indicate that exposure to SARS-CoV-2 S protein also has a predominant prolonged impact on a subset of differentially expressed HERV loci, leading to additional HERV activation in later stages of SARS-CoV-2 infection [97]. This specific SARS-CoV-2 S protein’s ability to modulate HERV expression, due to HERVs’ numerous interindividual variations, may also be the main reason for the unique interindividual COVID-19 immune signature. Onwards, in patients with post-acute long COVID syndrome, significantly higher levels of anti-SARS-CoV-2 S IgG, IgA, but also IgE antibodies were observed. Furthermore, exceptionally high serum levels of IgE as opposed to N, M and S proteins appeared to be significantly correlated with the degradation of physical functions in long COVID syndrome and disease severity. IgE, as a classical mast cell activator, triggers these cells to release histamine along with a variety of proinflammatory chemokines and cytokines, TNF-α, IL-1β and IL-6 included. Since IgE antibodies are known to be responsible for immunoallergic conditions and antiparasitic humoral reactions, this abnormal anti-SARS-CoV-2 S IgE response, i.e., the implication of mastoid cells in the onset of intense hyperinflammatory reaction and sustentation of “cytokine storm”, in these patients once again points to an unusual dysregulation of both cellular and humoral immunity in long COVID syndrome. The IgE role in the disease onset also explains the positive results of IgE blocker omalizumab and corticosteroids in the treatment of severe COVID-19 [73,98,99].

## 7. Conclusive Remarks

In summary, the innate immune response is central to the clearance of SARS-CoV-2, and studies show a clear role of effective immune response in protection from COVID-19 [82]. Part of the immune response is also underlined by neutralizing antibodies against the S protein. The licensed COVID-19 vaccines were developed as genetic products aimed to deliver a gene construct into the body for the production of the recombinant S protein. A large percentage of these products were mRNA-based or were based on replication-incompetent adenovirus vectors encoding full-length S protein with two amino acids in the S2 subunit mutated into proline [82,100]. The products are meant for intramuscular injection, where an IgG response is expected, although lower levels of IgM and IgA are induced by mRNA-based products as well [101]. However, recently documented specific activation of IgG4 upon these gene constructs injections has been described in the context of the immune tolerance mechanism to the S protein and suppression of the innate antiviral response. Importantly, the IgG4 levels upon repeated mRNA vaccination with high antigen concentrations have been implicated in the induction of autoimmune diseases and cancer [102]. Upon inoculation of the gene constructs, both T cell and B cell responses versus the S protein were measured, and antibody levels were comparable with those in convalescents [103]. Intriguingly, vaccine-evoked antibodies are more directed to the RBD than the antibodies evoked by natural infection [104]. Considering these observations about the important role of natural antiviral response towards SARS-CoV-2, and in particular S1 effects alone, as sufficient to advocate cell signaling cascade and dysregulation of natural and adaptive immunity response in certain individuals, recent data on adverse long-term effects in the vaccinated population are not surprising. Tissue damage and disruption of the immunological mechanisms may underlie some observed long-term health effects in the COVID-19-vaccinated population. For example, neurological disorders have been documented upon vaccination [105]. Moreover, knowing that IgG4 may impair the production of the antibodies IgG1 and IgG3 involved in cancer surveillance, the correlation between COVID-19 vaccination and observed fast-growing cancers in the general population has a strong scientific rationale [106,107,108,109]. Last year, accordingly, Dr. Wiseman and four other experts prepared a comprehensive document for the National Academies Committee reviewing relevant adverse events associated with COVID-19 vaccines, which will not be elaborated here in detail. The readers may refer to the document for deeper insight [110]. One aspect, however, relevant for the malignant transformation of cells we want to point out herein, is the possible correlation of transposable elements' role and impairment of the DNA repair mechanisms, particularly their role in underpinning additional DNA damage in the context of HERV-K integrase expression [50,111,112]. While summarizing the molecular effects of COVID-19 vaccines, it should be, however, emphasized that the majority of approved vaccines were developed to use the complete S glycoprotein sequence with modified structural characteristics aimed to improve the protein expression and immunogenicity while others used the approach to use highly immunogenic regions within the S glycoprotein, such as the RBD. Accordingly, differences in final molecular effects in vivo may be expected [113].

Taken together, all of these findings indicate that activation of transposable HERV elements triggered by SARS-CoV-2 S protein and/or SARS-CoV-2 infection of cells is observed. This phenomenon is observed along with a consequent natural immunity-related gene induction. In particular, the release of cytokines TNF-α, IL-1β and IL-6 occurs, which plays a notable role in sustaining chronic inflammation. Moreover, HERVs’ well-known interindividual variations might answer some open questions about the interpersonal variability in COVID-19 symptoms as well as unwanted events post-vaccination [114]. This makes a consecutive base for further studies in order to clarify the SARS-CoV-2 S protein-specific cellular and molecular ways of triggering HERVs, and particularly how exactly and which HERV ENVs dysregulate natural and adaptive immunity as a response to the SARS-CoV-2 protein.
